# The acceptance to heterologous booster vaccination of COVID-19 vaccine among HCWs and targeted population: A cross-sectional study in central China

**DOI:** 10.3389/fpubh.2022.943876

**Published:** 2022-08-01

**Authors:** Wei Qin, Xiaqing Zhang, Yao Wang, Fan Pan, Kai Cheng, Fangfang Huang, Jian Song, Hong Su

**Affiliations:** ^1^Department of Epidemiology and Health Statistics, School of Public Health, Anhui Medical University, Hefei, China; ^2^Department of Expanded Program on Immunization, Lu'an Municipal Center for Disease Control and Prevention, Lu'an, China; ^3^Department of Health Inspection and Quarantine, School of Public Health, Anhui Medical University, Hefei, China; ^4^Department of Nutrition and Food Hygiene, School of Public Health, Anhui Medical University, Hefei, China; ^5^Department of Preventive Medicine, School of Public Health, Bengbu Medical College, Bengbu, China

**Keywords:** COVID-19, willingness, heterologous booster vaccination, healthcare workers (HCWs), acceptance

## Abstract

**Background:**

There are few studies reported on the acceptance of heterologous booster vaccination for the COVID-19 vaccine among healthcare workers (HCWs) and the general population. We aimed to address that gap and explore determinant factors of acceptance of the heterologous booster vaccination.

**Methods:**

We conducted a cross-sectional study to examine the prevalence and determinant factors of the acceptance of heterologous booster vaccination for the COVID-19 vaccine among HCWs and the targeted population.

**Results:**

A total of 364 HCWs and 1,898 targeted populations were investigated in our study. 76.4% HCWs would recommend heterologous booster vaccination to their patients and 59.8% targeted population endorsed a clear willingness to receive this strategy. Compared with the adenoviral vector vaccine (AD5-nCOV), recombinant protein vaccine (ZF2001) was more preferred by HCWs (79.1%) and the targeted population (72.0%) as a heterologous booster vaccine. HCWs who did not work in the vaccination clinics were more likely to recommend heterologous booster vaccination (OR = 3.3, CI: 1.5–7.3). The targeted population aged 18–59 years (OR = 1.5, 95% CI:1.1–2.3), had a positive attitude toward COVID-19 vaccination (OR = 3.8, 95% CI: 1.7–8.6), had confidence in the safety of COVID-19 vaccines (OR = 6.6, 95% CI: 4.2–10.2), followed the recommendation of HCWs (OR = 33.6, 95% CI: 22.0–51.2), took initiative in collecting booster shots information (OR = 2.1, 95% CI: 1.5–3.0), and were familiar with the heterologous strategy (OR = 1.9, 95% CI: 1.1–3.1) were more likely to choose heterologous booster vaccination. The history of side effects of inactivated COVID-19 vaccine was a negative factor in choosing heterologous booster vaccination (OR = 0.4, 95% CI: 0.4–1.0).

**Conclusions:**

The heterologous booster vaccination strategy on the COVID-19 vaccine could be widely accepted among HCWs, whereas its acceptance among targeted population was only moderate. Public authorities should make efforts to communicate the public about the effectiveness and safety of the heterologous booster vaccination which could help increase their willingness to get vaccinated.

## Introduction

The global pandemic of coronavirus disease 2019 (COVID-19) caused by severe acute respiratory syndrome coronavirus 2 (SARS-CoV-2) has lasted more than 2 years and caused tremendous losses to the world (1). Scientists all over the world have made quick and unprecedented efforts to develop vaccines for ending the pandemic. In record time, several different types of vaccines were produced in both developed and developing countries, including mRNA vaccine, inactivated vaccine, adenoviral vector vaccine, and recombinant protein vaccine (2). As of 10 May 2022, more than 11.6 billion vaccine doses have been administered globally (3). There is no denying that vaccination has a commendable expectancy in preventing COVID-19-related severe disease, hospitalizations, and death (4, 5). However, the performance of these COVID-19 vaccines in preventing infection appears to be frustrating and breakthrough infection cases have been increasing (6–8). This is possibly related to the continuous decline of neutralization titer over time in vaccinated individuals and the emergence of SARS-CoV-2 variants such as Delta and Omicron (9–11). There is no doubt this has brought enormous challenges to the global response to the COVID-19 pandemic.

Existing studies reported that booster vaccination showed a higher immune response and may provide a way to control COVID-19 transmission without costly social-distancing measures and quarantines (12–14). In terms of fighting the emerging SARS-CoV-2 variants and waning vaccine-induced immunity, some countries, such as the UK (15), the US (16), and Israel (17) recommend their citizens receive booster shots. This strategy was not without controversy, particularly given the continued unequal access to vaccinations worldwide (18, 19). Nonetheless, the strategic rationale behind this strategy was that widespread uptake of COVID-19 booster vaccines in the general population could help to prevent further deaths and serious illness, and reduce the burden on the healthcare system (15). There are two options for the booster shots among fully vaccinated persons, including homologous boosters (same as the primary vaccine) and heterologous boosters (different from the primary vaccine) (20). Atmar et al. reported that administration of both homologous and heterologous booster vaccines had an acceptable safety, whereas heterologous boosters provided similar or higher neutralizing antibody titer levels (20). Similar results were observed in the heterologous prime-boost regimens with the inactivated vaccines and the adenoviral vector vaccine or recombinant protein subunit vaccine (21, 22). Therefore, we believe that the heterologous booster vaccination strategy may be valuable in reducing the disease burden of the global Omicron pandemic.

China is widely acknowledged as one of the countries with the smoothest vaccination programs and has begun to implement booster shots for adults over 18 years in October 2021 (23). However, the immunization strategy of the booster vaccination has been constantly adjusted, from only homologous vaccination at the early stage (from October 2021 to February 2022) to voluntary selection of homologous or heterologous vaccination at present. Therefore, understanding the acceptance of the heterologous booster vaccination strategy is critical for the adequate supply of COVID-19 vaccines and for helping inform public health authorities about what types of intervention measures are necessary to achieve broader community uptake. Vaccination willingness and trust in vaccines are the key factors for the success of any vaccination campaign (24, 25). Several studies have investigated willingness to receive the booster dose of the COVID-19 vaccine and found that the determinants of acceptance differed by study region (15, 26, 27). However, there are few studies reported on the acceptance of heterologous booster vaccination of COVID-19 vaccine among healthcare workers (HCWs) and the general population. Therefore, this study aimed to address that gap and explore determinant factors of acceptance of the heterologous booster vaccination.

## Methods

### Study setting

This survey was conducted in Lu'an which is a typical inland city located in the central region of China. It has the highest total area of 15,451.2 square kilometers in Anhui Province and about 4.39 million permanent residents. In March 2021, Lu'an launched a mass vaccination campaign for the COVID-19 vaccine. By the end of December 2021, 3.6 million targeted population (>3 years old) have fully vaccinated with the last dose of the primary series and we began to arrange booster shots for adults over 18 years. According to the guidelines of the National Health Commission of China, only the homologous booster vaccination strategy was adopted in Lu'an at the first stage of booster shots (October 2021–February 2022). By the end of February 2022, the strategy of heterologous booster vaccination was put on the agenda in Lu'an, and our study was carried out before it was officially promoted.

### Definitions

The homologous and heterologous COVID-19 booster vaccinations in this study were defined as follows: (1) the homologous COVID-19 booster vaccination was defined as three doses of inactivated COVID-19 vaccines (Sinopharm vaccine and Sinovac CoronaVac); (2) the heterologous COVID-19 booster vaccination was defined as two doses of inactivated COVID-19 vaccines (Sinopharm vaccine and Sinovac CoronaVac) plus one dose of adenoviral vector vaccine (AD5-nCOV) or plus one dose of recombinant protein vaccine (ZF2001).

### Study design and data collection

We divided this study into two parts according to the protocol, including a survey on the willingness to recommend heterologous booster vaccination to their patients among HCWs (survey I) as well as on the willingness to accept it among the targeted population aged more than 18 years (survey II). To avoid the potential effects of the two parts on each other, the two surveys were performed separately at different periods.

Between 21 and 27 February 2022, we conducted a cross-sectional questionnaire-based survey (survey I) to examine self-reported willingness to recommend the heterologous booster vaccination among staff from all 159 vaccination clinics in Lu'an. The self-administered questionnaire was distributed to each vaccination clinic. We considered that the participants must be department heads, doctors, nurses, and other staff who were directly engaged in the COVID-19 vaccination. We calculated the sample size by using the followed formula as follow: $n={\left(\frac{\mu_{1-\alpha /2}}{\delta }\right)}^{2}\;\times$*p* × (1–*p*), where the statistic value of μ_1−α/2_ is 1.96 at 95% confidence interval (CI), the δ represent the admissible error (δ = 5%), and the *p* represent the prevalence of the willingness to recommend COVID-19 vaccination which was estimated to be 79.6% in previous study (28). Finally, we calculated that a minimum sample size was 255 persons. Therefore, we also required that at least two persons should be surveyed in each clinic to meet minimum sample size requirements. After the deadline, a total of 364 HCWs completed the questionnaire. To ensure the quality of the survey, each questionnaire was checked for logical rationality by staff from Lu'an CDC.

**Figure 1:**
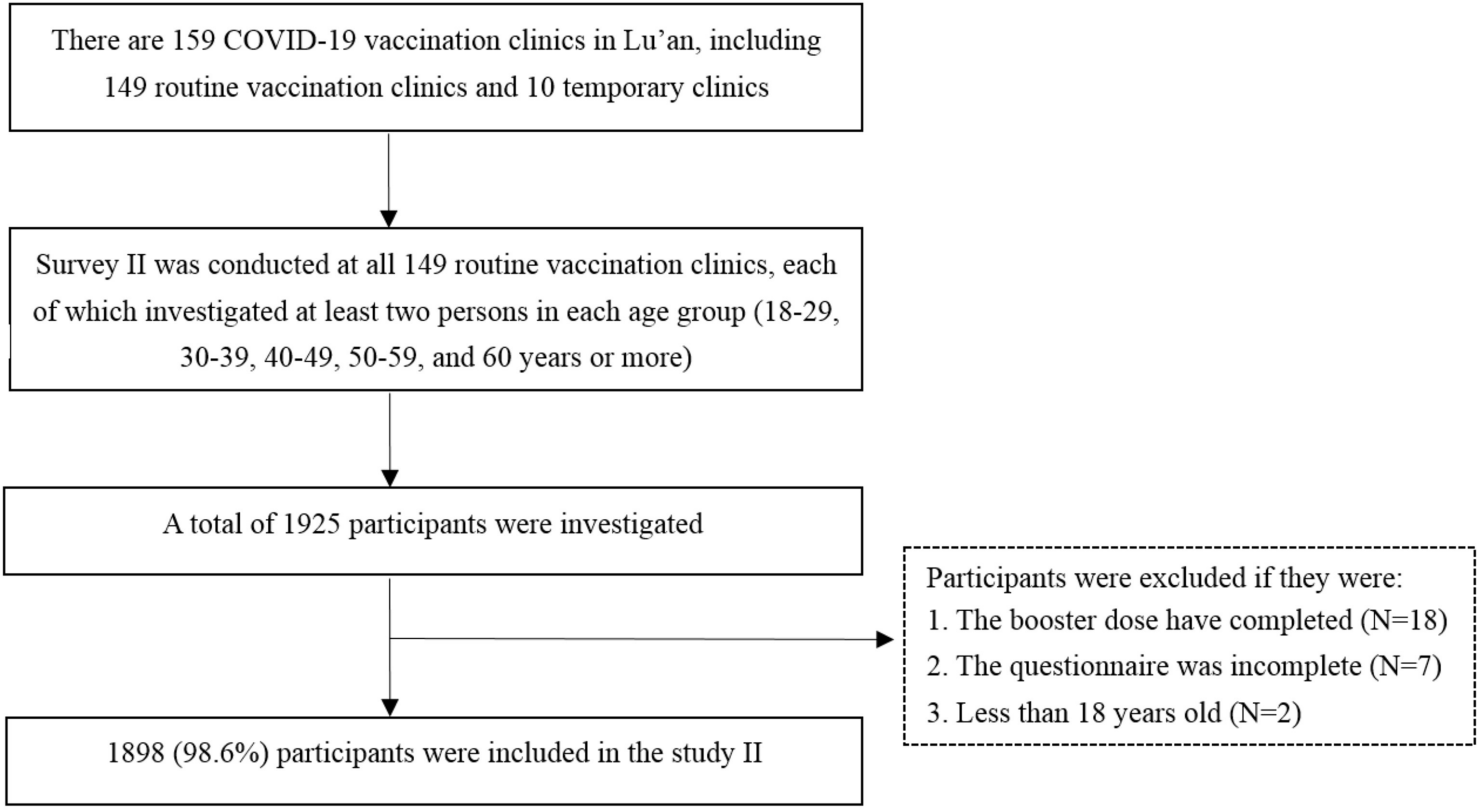
The flowchart of participant selection in survey II.

Between 1 and 15 March 2022, we launched another cross-sectional study (survey II) among the targeted population to assess the willingness to receive the heterologous booster vaccination and its determinants. We calculated the sample size by using the same formula as survey I. Since there was no published study for reference, we assumed that 50% of the respondents were willing to accept the heterologous booster vaccination (*p* = 50%). Finally, we calculated that a minimum target sample size of 385 was required. The inclusion criteria for the participants of survey II were as follows: (1) adults aged more than 18 years; (2) people who have received two doses of inactivated COVID-19 vaccines (Sinopharm vaccine and Sinovac CoronaVac); (3) people who have not received any booster shots. Those participants who could not be verified vaccination status or whose questionnaires were incomplete were excluded from the study. This survey was carried out by vaccination clinic doctors who have been trained by the local Center for Disease Control and Prevention (CDC). There are 159 COVID-19 vaccination clinics in Lu'an, including 149 routine vaccination clinics and 10 temporary clinics which are set in factories or large communities. Since the high mobility of staff from temporary clinics, we only rely on the routine vaccination clinics to carry out this survey to reduce the information bias. We designed a structured questionnaire that was sent to 149 routine vaccination clinics. Since the targeted population in our study were those who have received two doses of inactivated COVID-19 vaccines, their general information (such as date of birth and immunization history, etc.) have been included in the Anhui Immunization Information Management System (AIIMS). Therefore, investigators of each vaccination clinic could screen a list of potential responders who meet the inclusion criteria from the AIIMS. At least 10 convenient samples from the AIIMS were investigated in each clinic for the survey *via* telephone or face-to-face. Although this study was based on convenience sampling, we have promoted the number of participants in the five age groups (18–29, 30–39, 40–49, 50–59, and ≥60 years) to reduce the bias, with no <2 persons in each age group. Besides, we implemented the following three methods to ensure the quality of the survey: (1) developed a detailed and unified protocol for the field investigation; (2) doctors who carried out the investigation have been trained by CDCs; (3) 10% of the questionnaires stratified by county were randomly selected by staff from Lu'an CDC after the deadline to determine if they met the inclusion criteria. If an unqualified questionnaire was identified, all the questionnaires from this clinic will be re-checked. Finally, 1,898 participants with valid data were included in survey II. The flowchart of participant selection and sample saturation monitoring procedures is shown in Figure 1.

### Questionnaires and measurement

The questionnaires used in this study were adapted from the instrument contents on vaccine acceptance of previous studies that have been published (25, 27, 28). We implemented a pilot survey at an urban clinic and a rural clinic to evaluate the comprehension of the questions and answers. We interviewed five HCWs and 19 potential participants during the period of the pilot survey and revised questionnaires based on the interview results. Data from the pilot survey was not included in the results.

The questionnaire of the survey I consisted of 11 questions to explore three domains relating to the HCWs: (1) demographic characteristics, including age, gender, education level, professional title, job position, years of work experience, location of clinics, etc.; (2) reasons for recommending and refusing to recommend the heterologous booster vaccination; (3) types of the heterologous booster vaccination. HCWs' willingness to recommend heterologous booster vaccination was measured using the question: “Would you like to recommend heterologous booster vaccination to your patients?”. The response options were willing, unwilling, and unsure. The last two options were merged into unwillingness during the final analysis.

For survey II, the major structure of the questionnaire was composed of 20 close-ended items that were divided into four sections. The first section was the sociodemographic characteristics, including age, gender, education level, residence, name of clinics, and type of investigation. The second section was COVID-19 vaccine-related questions, including the history of COVID-19 vaccination, vaccine type, the number of doses, etc. The third section was potential drivers of heterologous booster vaccination acceptance or rejection, including perceptions of the importance of administration with COVID-19 vaccine, history of side effects following inactivated vaccines, confidence in the safety of COVID-19 vaccines, and knowledge of heterologous booster vaccination. The fourth section had a set of reasons for accepting as well as refusing heterologous booster vaccination. Participants' willingness to receive heterologous booster vaccination was measured using the question, “Would you like to accept heterologous booster vaccination when you take the booster shot?”. The response options were willing, unwilling, and unsure. The last two options were merged into unwillingness during the final analysis.

### Data analysis

All collected data were entered into Microsoft Excel 2016. We used IBM SPSS Statistics for Windows version 20.0 (IBM Corp, Armonk, NY, USA) to perform data cleaning and statistical analysis. Categorical variables are expressed as count and percentage in different groups. The univariable logistic regression model was performed to explore the factors associated with the willingness to recommend heterologous booster vaccination among HCWs. The chi-squared test was used to initially estimate differences in variables between participants with and without the willingness to receive heterologous booster vaccination in survey II. The multivariate logistic regression model was used to explore the factors associated with the willingness to receive a heterologous booster vaccination. The odds ratio (OR) corresponding and 95% CI were calculated. Two-sided *p*-values were reported to be statistically significant at <0.05.

## Results

### Characteristics of HCWs

A total of 364 HCWs were recruited in the survey I. The characteristics and their willingness to recommend the heterologous booster vaccination against COVID-19 are summarized in Table 1. The median age was 40 (interquartile range: 31–48) years, and 65.4% of them were female. The median work experience years was 6.5 (interquartile range, 2–14) years. About 81.6% HCWs with a junior college or more education level, and 70.6% with a junior professional title. 79.1% of them were staff or heads of vaccination clinics, and 20.9% worked in other departments of the hospital.

**Table 1:** Characteristics of HCWs and their willingness to recommend the heterologous booster vaccination against COVID-19 (*N* = 364).

**Variables**	**Total sample *N* (%)**	**Willingness to recommend heterologous**		***P*-value**	**OR (95 CI %)**
		**booster vaccination**			
		**Yes, *n* (%)**	**No/unsure, *n* (%)**		
**Gender**					
Male	126 (34.6)	102 (81.0)	24 (19.0)	0.135	1.5 (0.9–2.5)
Female	238 (65.4)	176 (73.9)	62 (26.1)		Reference
**Age (yr)**					
19–39	181 (49.7)	133 (73.5)	48 (26.5)	0.196	0.7 (0.4–1.2)
≥40	183 (50.3)	145 (79.2)	38 (20.8)		Reference
Education level					—
Technical school or below	67 (18.4)	53 (79.1)	14 (20.9)	0.593	1.2 (0.6–2.5)
Junior college	161 (44.2)	122 (75.8)	39 (24.2)	0.993	1.0 (0.6–1.7)
Bachelor's degree or above	136 (37.4)	103(75.7)	33(24.3)		Reference
**Professional title**					
Junior	257 (70.6)	197 (76.7)	60 (23.3)	0.132	0.2 (0.03–1.82)
Middle	92 (25.3)	67 (72.8)	25 (27.2)	0.086	0.19 (0.2–1.5)
Senior	15 (4.1)	14 (93.3)	1 (6.7)		Reference
**Job position**					
HCWs in other departments	76 (20.9)	68 (89.5)	8 (10.5)	0.002	3.3 (1.5–7.3)
Head of vaccination clinic	85 (23.3)	64 (75.3)	21 (24.7)	0.557	1.2 (0.7–2.1)
Staff of vaccination clinic	203 (55.8)	146 (71.9)	57 (28.1)		Reference
**Years of work experience**					
<5	143 (39.3)	107 (74.8)	36 (25.2)	0.562	0.8 (0.5–1.5)
5–9	99 (27.2)	76 (76.8)	23 (23.2)	0.846	0.9 (0.5–1.8)
≥10	122 (33.5)	95 (77.9)	27 (22.1)		Reference
**County**					
Yu'an	70 (19.2)	65 (92.9)	5 (7.1)	<0.001	42.0 (13.4–131.5)
Jinzhai	51 (14.0)	45 (88.2)	6 (11.8)	<0.001	29.1 (9.2–92.1)
Shucheng	44 (12.1)	37 (84.1)	7 (15.9)	<0.001	20.5 (6.7–62.8)
Huoqiu	70 (19.2)	58 (82.9)	12 (17.1)	<0.001	18.7 (6.9–50.7)
Jin'an	76 (20.9)	59 (77.6)	17 (22.4)	<0.001	13.4 (5.2–34.6)
Yeji	14 (3.9)	6 (42.9)	8 (57.1)	0.104	2.9 (0.8–10.8)
Huoshan	39 (10.7)	8 (20.5)	31 (79.5)		Reference

### Willingness to recommend heterologous booster vaccination and its influencing factors

Among 364 HCWs, 278 (76.4%) responded that they would recommend the heterologous booster vaccination to their patients, 65 (17.8%) would not recommend it, and 21 (5.8%) would hesitate to recommend it. The results of the univariable logistic regression model were shown in Table 1. There was a large disparity in willingness to recommend heterologous booster vaccination among HCWs from different counties, where Yu'an district was the highest (92.9%) and Huoshan county was the lowest (20.5%). Furthermore, we found that HCWs who did not work in the vaccination clinics were more likely to recommend heterologous booster vaccination (OR = 3.3, CI: 1.5–7.3).

### Characteristics of the targeted population

A total of 1,898 participants were included in survey II, of which 1,282 (67.5%) were investigated face-to-face. Table 2 presents the characteristics of participants and their acceptance of heterologous booster vaccination. A total of 1,002 (52.8%) participants were male; 1,540 (81.1%) were rural residents and 358 (18.9%) were urban residents; the median age was 46 (interquartile range: 32–58) years. Regarding their educational status, most of the participants (80.2%) were in senior high school or below, and only 375 (19.8%) had accomplished college or above education.

**Table 2:** Characteristics of respondents and their acceptance of heterologous booster vaccination against COVID-19 (*N* = 1,898).

**Variables**	**Total sample** ***N* (%)**	**Heterologous booster vaccination acceptance**		**χ^2^ value**	***P*-value**
		**Yes, *n* (%)**	**No/unsure, *n* (%)**		
**Type of investigation**				9.836	**0.002**
Face to face	1,282 (67.5)	798 (62.3)	484 (37.7)		
Telephone	616 (32.5)	337 (54.7)	279 (45.3)		
**Age (y)**				7.355	0.007
18–59	1,473 (77.6)	905 (61.4)	303 (38.6)		
≥60	425 (22.4)	230 (54.1)	195 (45.9)		
**Gender**				0.296	0.586
Male	1,002 (52.8)	605 (60.4)	397 (39.6)		
Female	896 (47.2)	530 (59.2)	366 (40.8)		
**Education level**				5.953	0.015
Senior high school or below	1,523 (80.2)	890 (58.4)	633 (41.6)		
College or above	375 (19.8)	245 (65.3)	130 (34.7)		
**County**				143.649	<0.001
Huoqiu	330 (17.4)	226 (68.5)	104 (31.5)		
Huoshan	191 (10.1)	74 (38.7)	117 (61.3)		
Jin'an	228 (12)	114 (50.0)	114 (50.0)		
Jinzhai	262 (13.8)	180 (68.7)	82 (31.3)		
Shucheng	554 (29.2)	329 (59.4)	225 (40.6)		
Yeji	103 (5.4)	29 (28.2)	74 (71.8)		
Yu'an	230 (12.1)	183 (79.6)	47 (20.4)		
**Residence**				4.683	0.03
Urban	358 (18.9)	196 (54.7)	162 (45.3)		
Rural	1,540 (81.1)	939 (61.0)	601 (39.0)		
**Time since administration with the last dose of inactivated COVID-19 vaccine**				0.492	0.483
<6 months	154 (8.1)	88 (57.1)	66 (42.9)		
More than 6 months	1,744(91.9)	1,047 (60.0)	697 (40.0)		
**Positive attitude toward COVID-19 vaccination**				59.423	<0.001
Yes	1,810 (95.4)	1,117 (61.7)	693 (38.3)		
No	88 (4.6)	18 (20.4)	70 (79.6)		
**History of side effects following inactivated COVID-19 vaccines**				11.081	0.001
Yes	51 (2.7)	19 (37.3)	32 (62.7)		
No	1,847 (97.3)	1,116 (60.4)	731(39.6)		
**Confidence in the safety of COVID-19 vaccines**				817.703	<0.001
Yes	1,361 (71.7)	1,089 (80.0)	272 (20.0)		
No	550 (29.0)	56 (10.2)	494 (89.8)		
**Familiar with heterologous booster vaccination**				165.488	<0.001
Yes	325 (17.1)	260 (80.0)	65 (20.0)		
Heard about it	782 (41.2)	536 (68.5)	246 (31.5)		
No	791 (41.7)	339 (42.9)	452 (57.1)		
**Take initiative in collecting booster shots information**				197.405	<0.001
Yes	841 (44.3)	652 (77.5)	189 (22.5)		
No	1,057 (55.7)	483 (45.7)	574 (54.3)		
**Accepting recommendations from HCWs**				10,69.929	<0.001
Yes	1,276 (67.2)	1,091 (85.5)	185 (14.5)		
No	622 (32.8)	44 (7.1)	578 (92.9)		

### Willingness to receive the heterologous booster vaccination

In all, 1,135 (59.8%) participants endorsed a clear willingness to receive the heterologous booster vaccination. A total of 11 statistically significant variables in the initial analysis were included in the multivariate analysis. The results of multivariate logistic regression analysis showed that face-to-face survey (OR = 2.0, 95% CI: 1.4–2.8), participants aged 18–59 years (OR = 1.5, 95% CI: 1.1–2.3), positive attitude toward COVID-19 vaccination (OR = 3.8, 95% CI: 1.7–8.6), confidence in the safety of COVID-19 vaccines (OR = 6.6, 95% CI: 4.2–10.2), following the recommendation of HCWs (OR = 33.6, 95% CI: 22.0–51.2), taking initiative in collecting booster shots information (OR = 2.1, 95% CI: 1.5–3.0), and familiar with heterologous booster strategy (OR = 1.9, 95% CI: 1.1–3.1) were positive factors associated with willingness to receive the heterologous booster vaccination (Table 3). The history of side effects of inactivated COVID-19 vaccine was a negative factor (OR = 0.4, 95% CI: 0.4–1.0). Interestingly, we also found large disparities in willingness to receive heterologous booster vaccination among different counties, where the rate of acceptance ranged from 28.2 to 79.6% (Table 2).

**Table 3:** Multiple logistic regression of factors associated with willingness to accept the heterologous booster vaccination.

**Variables**	**β**	**S.E**.	**Wald χ^2^**	***P-*value**	**OR (95% CI)**
**Type of investigation**					
Face to face	0.672	0.178	14.283	<0.001	2.0 (1.4–2.8)
Telephone					Reference
**Age (y)**					
18–59	0.433	0.193	5.025	0.025	1.5 (1.1–2.3)
≥60					Reference
**Education level**					
Senior high school or below	0.374	0.208	3.234	0.072	1.5 (1.0–2.2)
College or above					Reference
**County**					
Huoshan	0.657	0.398	2.723	0.099	1.9 (0.9–4.2)
Jinan	1.098	0.386	8.077	0.004	3.0 (1.4–6.4)
Shucheng	1.876	0.355	27.866	<0.001	6.5 (3.3–13.1)
Huoqiu	1.651	0.381	18.743	<0.001	5.2 (2.5–11)
Jinzhai	1.089	0.371	8.633	0.003	3.0 (1.4–6.1)
Yuan	1.835	0.405	20.56	<0.001	6.3 (2.8–13.9)
Yeji					Reference
**Residence**					
Urban	0.265	0.212	1.56	0.212	1.3 (0.9–2.0)
Rural					Reference
**Positive attitude toward COVID-19 vaccination**					
Yes	1.339	0.412	10.542	0.001	3.8 (1.7–8.6)
No					Reference
**History of side effects following inactivated COVID-19 vaccines**					
Yes	−0.892	0.453	3.883	0.049	0.4 (0.2–1.0)
No					Reference
**Confidence in the safety of COVID-19 vaccines**					
Yes	1.881	0.225	69.609	<0.001	6.6 (4.2–10.2)
No					Reference
**Familiar with heterologous booster vaccination**					
Yes	0.624	0.257	5.883	0.015	1.9 (1.1–3.1)
Heard about it	0.784	0.179	19.225	<0.001	2.2 (1.5–3.1)
No					Reference
**Taking initiative in collecting booster shots information**					
Yes	0.761	0.175	18.833	<0.001	2.1 (1.5–3.0)
No					Reference
**Accepting recommendations from HCWs**					
Yes	3.514	0.215	267.074	<0.001	33.6 (22.0–51.2)
No					

### Reasons for accepting and refusing heterologous booster vaccination

**Figure 2:**
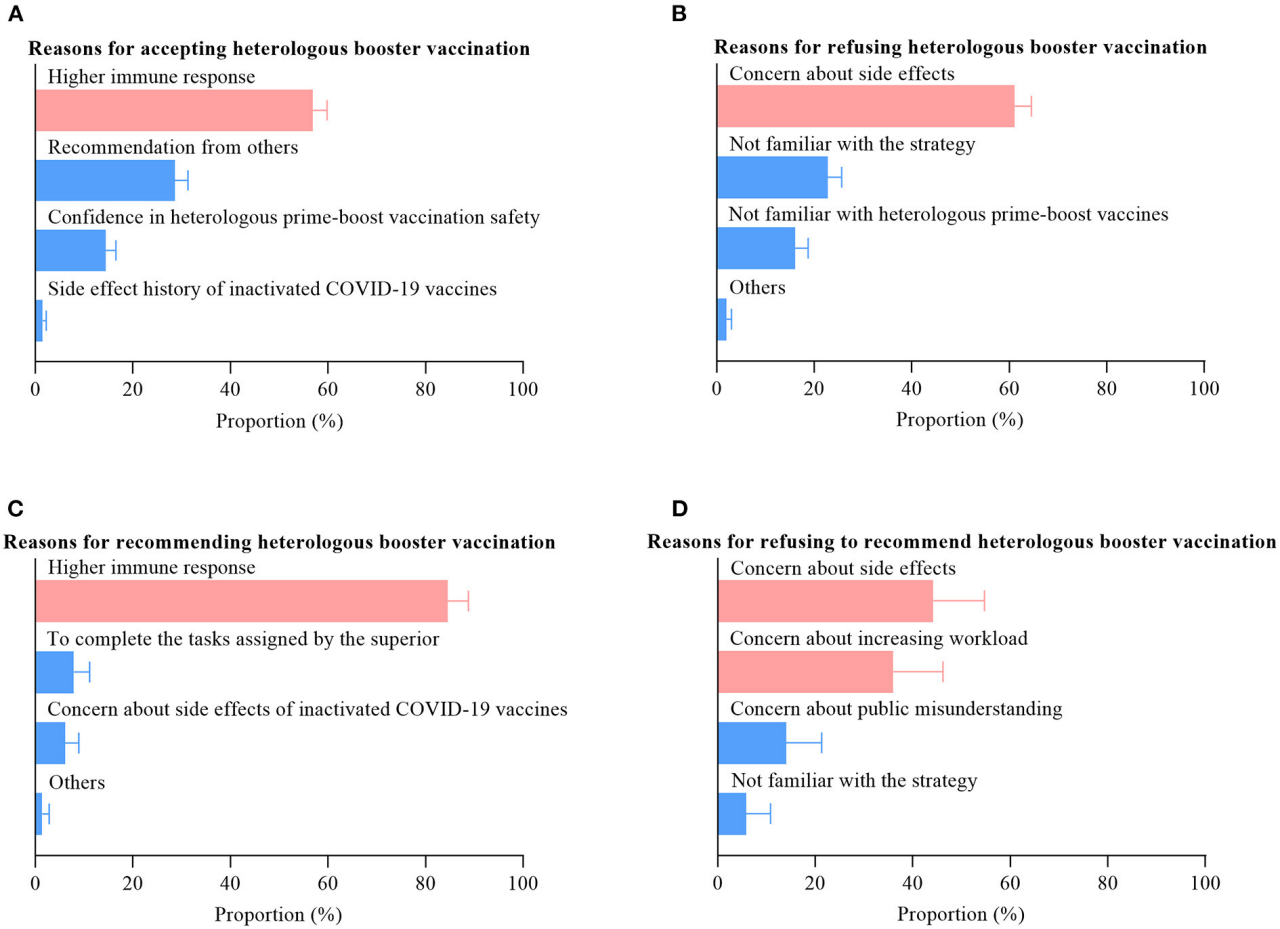
Reasons for accepting or refusing heterologous booster vaccination.

The most prevalent reason for accepting heterologous booster vaccination among the targeted population was the perception of a higher immune response (56.9%, Figure 2A), whereas the primary reason for refusing was concern about side effects (61.1%, Figure 2B). We observed similar results among HCWs (Figures 2C,D). Moreover, we also found that another important reason for refusing to recommend heterologous booster vaccination among HCWs was concern about increasing workload (36.0%).

### Vaccine selection for the heterologous booster vaccination

Our results showed that ZF2001 was the first choice for the heterologous booster vaccination among both HCWs and the targeted population. Among HCWs who are willing to recommend heterologous booster vaccination, 79.1% (220/278) will choose the strategy of inactivated COVID-19 vaccines plus ZF2001. Likewise, among the targeted population who are willing to receive heterologous booster vaccination, 72.0% (817/1135) will choose the strategy of inactivated COVID-19 vaccines plus ZF2001.

## Discussion

The current study explored the prevalence and determinant factors of the acceptance of heterologous booster vaccination for the COVID-19 vaccine among HCWs and the targeted population. Our findings indicated that 76.4% HCWs would recommend the heterologous booster vaccination to their patients and 59.8% of adults endorsed a clear willingness to receive the heterologous vaccine as the booster dose. We also found that participants followed the recommendation of HCWs (OR = 33.6, 95% CI: 22.0–51.2) and those who were confident in the safety of the COVID-19 vaccines (OR = 6.6, 95% CI: 4.2–10.2) are more likely to choose heterologous booster vaccination. Interestingly, we observed that HCWs who did not work in vaccination clinics prefer to recommend the heterologous booster vaccination (OR = 3.3, 95% CI: 1.5–7.3). Besides, our study indicated that ZF2001 was more preferred by HCWs and the targeted population as a heterologous booster vaccine, suggesting that we may give priority to ZF2001 compared with AD5-nCOV when supplying booster COVID-19 vaccines.

Existing studies support the evidence for booster dose efficacy against both Delta and Omicron variants (29, 30). Therefore, many countries have decided to push forward the booster vaccination to as many individuals as possible to achieve maximum public health benefits against the emerging SARS-CoV-2 variants (31). Identifying factors that influence booster vaccination acceptance will help to determine groups that will most readily accept a booster dose. Several studies have shown that the acceptance of the booster dose of the COVID-19 vaccine varied among different countries and regions. Qin et al. reported that 93.7% of responders were willing to receive a third dose of the COVID-19 vaccine in China (32). In a university community in Italy, 85.7% were willing to receive the booster dose (26). We found that most of the previous surveys were based on the population who would receive the homologous booster vaccination. However, the homologous booster vaccination is an existing common immunization strategy, while the heterologous booster vaccination is a new alternative strategy against COVID-19 in China. Therefore, the general population still needs an adaptation period for this new strategy, and its acceptance in particular needs to be evaluated.

Several studies indicated that HCWs' recommendation is one of the strongest influencers in vaccination decision-making among children and their parents (33, 34). In this study, we found that 76.4% HCWs would be willing to recommend the heterologous booster vaccination to their patients, which was broadly in agreement with the results (79.6%) from France (28). A principal reason for such attitudes seems to be the perception of a higher immune response with the heterologous prim-booster vaccination strategy among HCWs. Previous studies reported that heterologous booster vaccination induced higher antibody titers than homologous booster vaccination (20–22, 35). Besides, the heterologous boosting could elicit strong T cell responses, which improve the breadth of immunity and overcome the limitations of the individual vaccine platforms (21). The main reasons for unwillingness to recommend heterologous booster vaccination among HCWs were concern about the side effects (44.2%) and increasing workload (36.0%). Worrying about the side effects is a common cause for refusing to recommend (25, 27) while worrying about increasing workload is an interesting topic.

However, we believe that this reason is not an accident event in China, but may actually exist. For example, our findings showed that HCWs who did not work in the vaccination clinics were more likely to recommend heterologous booster vaccination (OR = 3.3, CI: 1.5–7.3). It might be explained that HCWs of vaccination clinics have to undertake a lot of routine immunization works in addition to the COVID-19 vaccination campaign, so they do not want to spend too much time explaining the heterologous booster vaccination strategy.

Currently, both heterologous and homologous booster vaccination strategies are being adopted in China, which means that the general population will have more options. Hence, it is crucial to understand the acceptance of heterologous booster vaccination and its determinant factors. We observed that 59.8% of participants endorsed a clear willingness to receive the heterologous booster vaccination, which was significantly higher than the results from Jordan (26.5%) (36). The most prevalent reason for accepting heterologous booster vaccination was the perception of a higher immune response (74.6%), which was consistent with the reason that HCWs choose to recommend this strategy. We also found large disparities in willingness to receive heterologous booster vaccination among different counties, where the rate of acceptance ranged from 28.2 to 79.6%. This means that we should not only balance the vaccine supply in different regions but also should conduct further investigations in low-acceptance regions to determine the specific influencing factors. Numerous studies indicated that people who were willing to accept the advice of HCWs were likely to be vaccinated (28, 33, 34). Therefore, public authorities should carry out training on this topic as soon as possible to address HCWs' concerns about the safety and immunogenicity of the heterologous booster vaccination. A meta-analysis showed that gender and education level were strong predictors of COVID-19 vaccination willingness (37). However, similar relationships were not observed in our study. The reason for this may be that the participants in this study were those who had already received two doses of inactivated COVID-19 vaccines.

### Strengths and limitations

Our study has some strengths. To the best of our knowledge, the willingness to receive the heterologous booster vaccination has never been evaluated in China. We aimed to address that gap and explore the determinant factors of acceptance of the heterologous vaccination among HCWs and the targeted population with booster vaccination. Second, survey II was based on face-to-face or telephone rather than online questionnaires, which means that the results may be closer to the real situation. As we all know, it is necessary to have direct communication with HCWs to obtain more useful information when people get vaccinated (34). Our findings are also subject to the following limitations. First, our investigation was only conducted in one city in China, so the findings may have limited generalizability. For example, we have found large disparities in willingness to receive heterologous booster vaccination even at the county level in Lu'an city. Second, the participants of survey II were only adults over 18 years and people who had not received booster shots, which means the acceptance prevalence in this study cannot represent that of general public. Third, this study is a cross-sectional survey and cannot show dynamic trends. Hence, we intend to follow up with the respondents after 3 months to observe the robustness of the results.

## Conclusion

In conclusion, our study provides important real-world evidence on the prevalence and determinant factors of the acceptance of the heterologous booster vaccination of the COVID-19 vaccines among HCWs and the targeted population. Our findings suggest that public authorities should make efforts to communicate to the HCWs and the public about the effectiveness and safety of the heterologous booster vaccination which could help increase public willingness to get vaccinated.

## Data availability statement

The raw data supporting the conclusion is with the first author and can be made available on reasonable request and prior approval.

## Ethics statement

The authors assert that all procedures contributing to this survey comply with the ethical standards of the relevant national and institutional committees on human experimentation and with the Helsinki Declaration. Also, all participants consented to participation in the survey verbally, and the data were kept confidential by all authors and participators.

## Author contributions

WQ designed, conceptualized this study, and drafted the manuscript. XZ and FH participated in the data clear and analysis. JS made constructive comments on data analysis and visualization. HS critically reviewed and supervised the development of the paper. YW, FP, WQ, and KC conducted the pilot survey, participated in the immunization record review, and quality control of the questionnaire. All the authors reviewed and edited the final manuscript.

## Funding

Our study was supported by the Special Funds for Expanded Program on Immunization in 2022 of the Lu'an municipal government.

## Conflict of interest

The authors declare that the research was conducted in the absence of any commercial or financial relationships that could be construed as a potential conflict of interest.

## Publisher's note

All claims expressed in this article are solely those of the authors and do not necessarily represent those of their affiliated organizations, or those of the publisher, the editors and the reviewers. Any product that may be evaluated in this article, or claim that may be made by its manufacturer, is not guaranteed or endorsed by the publisher.
